# A feasibility study to assess Mediterranean Diet adherence using an AI-powered system

**DOI:** 10.1038/s41598-022-21421-y

**Published:** 2022-10-11

**Authors:** Ioannis Papathanail, Maria F. Vasiloglou, Thomai Stathopoulou, Arindam Ghosh, Manuel Baumann, David Faeh, Stavroula Mougiakakou

**Affiliations:** 1 ARTORG Center for Biomedical Engineering Research, University of Bern, Murtenstrasse 50, Bern, 3008 Switzerland; 2 Oviva AG, Zürcherstrasse 64, Altendorf, 8852 Switzerland; 3 Epidemiology, Biostatistics and Prevention Institute (EBPI), University of Zurich, Zurich, 8001 Switzerland

**Keywords:** Nutrition, Weight management, Image processing, Machine learning

## Abstract

Mediterranean diet (MD) can play a major role in decreasing the risks of non-communicable diseases and preventing overweight and obesity. In order for a person to follow the MD and assess their adherence to it, proper dietary assessment methods are required. We have developed an Artificial Intelligence-powered system that recognizes the food and drink items from a single meal photo and estimates their respective serving size, and integrated it into a smartphone application that automatically calculates MD adherence score and outputs a weekly feedback report. We compared the MD adherence score of four users as calculated by the system versus an expert dietitian, and the mean difference was 3.5% and statistically not significant. Afterwards, we conducted a feasibility study with 24 participants, to evaluate the system’s performance and to gather the users’ and dietitians’ feedback. The image recognition system achieved 61.8% mean Average Precision for the testing set and 57.3% for the feasibility study images (where the ground truth was taken as the participants’ annotations). The feedback from the participants of the feasibility study was also very positive.

## Introduction

Adhering to the Mediterranean Diet (MD) has been proven to be beneficial against non-communicable diseases such as cardiovascular diseases, type 2 diabetes mellitus, and cancer^1,2^. Adherence to MD includes frequent consumption of vegetables, fruits, nuts, cereal, legumes, and olive oil and a lower intake of eggs, red/processed meat, and sweets^3^. The quantification of a person’s MD adherence (MDA) takes place on a weekly basis considering the person’s consumption of foods, taking into account both frequency of the food categories and their consumed amount. Such a set of rules has been defined by experienced dietitians/nutritionists in our previous work^4^.

To calculate the MDA score, an accurate and objective method of dietary assessment is of utmost importance. The first method created to assess the MDA score was published by Trichopoulou et al.^5–7^. The score consists of 9 components, each giving a score of 0 or 1, up to a maximum of 9 points. Beneficial components (vegetables, fruits, legumes, cereals, fish) give 1 point when the person’s consumption of these components was above the median value (different for men and women), while for meat and dairy products users would get 1 point for having consumed less than the median. People should also have moderate ethanol consumption, and high monounsaturated and polyunsaturated to saturated fat ratio, in order to get two more points. Another study^8^ recommended using 13 MD food groups where each group gives a score of 0–10, based on the daily consumption of this food. The score is then standardized to lie between 0 and 100. A 14-question MDA screener was also created^9^, consisting of questions related to the frequency of consumption of 12 food items, if the person is using olive oil for cooking, and if they prefer white meat over red meat. However, traditional dietary assessment methods, such as food frequency questionnaires and 24-h recall are time-consuming and prone to errors due to subjective estimation of a meal’s serving size.

In recent years, with the development of machine learning (ML) and computer vision algorithms, a plethora of automatic dietary assessment systems has emerged. A survey of potential nutrition-app users mentioned that incorrect calorie and nutrient estimations are a reason for not selecting an app^10^. In a review of 22 different innovative dietary assessment systems, it was concluded that end-users, including healthcare professionals (HCPs), need to participate in the design and development of such solutions contributing to the several technical challenges and research questions that need to be answered^11^. Indeed, 1001 HCPs (833 physicians, 75 dietitians, 62 nurses, 31 other) took part in a survey on nutrition apps^12^ which included questions regarding (i) the dietary assessment methods they use, (ii) the reasons for not being satisfied with a nutrition app, (iii) the reasons for recommending or not a nutrition app, (iv) the criteria, features, and barriers for selecting an app, and (v) preferences for the display of the results. 45.5% of the HCPs have recommended a nutrition app to their clients/patients and the most important criteria for selecting an app were if it was validated and if automatic food recording and automatic nutrient and energy estimation were supported.

The majority of the systems use a single meal image^13–15^ or a short video^16,17^ to perform automatic dietary assessment. Typically, the procedure consists of three steps: (i) food item segmentation, (ii) food recognition, and (iii) volume estimation. In a study with hospitalized patients, an AI-powered system recognizes and segments the different food categories that appear in each image^13,14^. The system estimates the nutritional intake of hospitalized patients, based on the segmented and recognized food items and the Red Green Blue—Depth (RGB-D) images before and after consumption. The goFOOD^TM^^16^ system detects and recognizes the food items that appear in a short video or two images. The system then reconstructs the 3D image, estimates the volume of the different food items, and automatically calculates their nutritional content, using food composition databases. The estimations of goFOOD^TM^ were compared to those of dietitians and the system outperformed the dietitians in a database consisting of European meals and had a similar performance in a fast-food dataset. Following our previous work^4^, to calculate the MDA score, we require only an approximation of the serving size of the food (e.g., 1 serving, 1 and a half servings), and not an accurate volume estimation (e.g., 100 ml). Since our goal was to integrate the system into a smartphone application, we wanted the system to be as user-friendly and easy-to-use as possible and require the minimum amount of effort from the user. Therefore, we used only a single RGB image to jointly perform food recognition and serving size estimation, as the food segmentation and food volume estimation tasks would require additional input from the users (two photos or a short video) and computational time.

In most realistic scenarios, each image contains multiple food items that have to be recognized (multi-label food recognition)^15–17^ as a first step in performing dietary assessment. To create a system that can automatically perform multi-label food recognition as well as serving size estimation with high accuracy, a dataset that contains labelled images is crucial. In practice, creating such a dataset with clean ground truth annotations of the food labels and their serving sizes requires the availability of expert annotators who have technical understanding of serving size estimation. This can be expensive in terms of time and effort. It is usually easier and less costly to collect more images and use crowdsourcing techniques to have them annotated by inexperienced annotators^18^. This, however, often leads to less accurate labelling of the images, leading to the creation of a dataset with label noise^19^.

In this paper we propose a novel end-to-end system, in the form of a smartphone application, that can automatically calculate the weekly MDA score of the users, based on individual images of their meals. The system uses AI-powered methods and algorithms to perform food image analysis by (a) recognizing all the food items that appear in a meal image using a Convolutional Neural Network (CNN), trained on food images, (b) estimating their serving sizes, and subsequently (c) using all the images of the week to calculate the automatic weekly MDA score. At the end of the week, the system generates and presents an automated feedback report to the user. This system has been validated in a feasibility study in the scope of the medipiatto (https://go-food.tech/medipiatto-balance-you-diet-improve-your-life/) research project. This is an extension of our previous work^4,20^ and the network has now been trained on a larger dataset that contains higher label noise. To our knowledge, this is the first end-to-end fully automatic system that jointly recognizes the food items and the serving sizes from a single image, calculates a compliance with a healthy diet score, and provides feedback and personalized suggestions to the user.

## Methods

### Database

In order to develop an AI system that successfully identifies food groups based on food images taken in everyday scenarios, we require a large number of appropriately annotated data. We created such a dataset, consisting of images that were captured under free-living conditions. A group of expert academic dietitians identified 31 categories of food items which are relevant for MDA calculation in our previous study^4^.

A group of 10 non-expert annotators was recruited to annotate the individual food items and their serving sizes in each food image. We chose to use serving sizes because it would be easier for a non-expert in dietetics to approximate using body parts (e.g. handful) or household measures (e.g. cup). We used the serving sizes as provided by British Nutrition Foundation^21^. Table 1 shows the amount of images that were annotated by a specific number of annotators.

**Table 1:** The number of images that were annotated by a specific number of annotators.

Number of annotators	Count of images
6	14
5	9104
4	770
3	394
2	598
1	144

e.g., we had 9104 images with 5 annotators each, and 770 with 4 annotators each.

**Figure 1:**
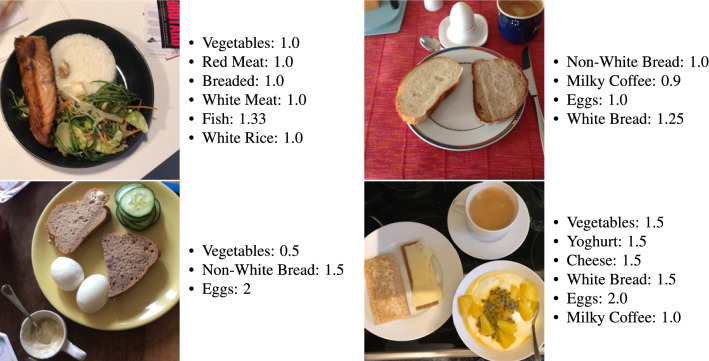
Example images of the training set (upper row) and the testing set (lower row) of the database, along with their annotations.

The annotators were provided with basic instructions, developed by experienced dietitians, for the annotation process on how to identify the 31 food categories and estimate the serving sizes. We collected a total of 11,024 images, along with their annotated labels and serving sizes. 9,888 of them have at least 4 annotators. Each image can contain a varying number of labels and the average number of labels for an image is 3.6. A subset of 293 images was taken as the testing set. An expert dietitian (i.e., with clinical experience, working for more than 5 years in the field) took over the refinement of the labels of the testing set, so that the system can be evaluated on a clean testing set. Some sample images from the training and the testing set can be seen in Fig. 1.

#### Inter annotator agreement metric

In this section, a new metric that quantifies the agreement of annotators both in terms of food recognition and serving size estimation is presented. Since the annotation was performed by non-expert annotators, the dataset contains natural label noise that we wanted to estimate. We used the Inter-Annotator Agreement (IAA) score for each of the 31 food categories, in order to quantify the degree of label noise in the dataset. However, since the labels of each image are not mutually exclusive (each image can contain more than one food item), IAA metrics such as Cohen’s and Fleiss’s kappa, which are frequently used in ML problems, could not be applied in our case. Moreover, we had to also consider the differences in the serving size estimations between the annotators. For these reasons we have rephrased the IAA score as follows: For every category *c* in each image, we calculate the normalized summation of the squared distances ($\tilde{ssd_{c}}$) between the estimated serving sizes by each annotator, and the IAA for a specific category as follows:

1 $$\begin{aligned} \tilde{ssd_{c}}= & {} \left( \dfrac{n(n-1)}{2}\right) ^{-1}\sum _{i=1}^{n}\sum _{j=i+1}^{n} (p_{ic} - p_{jc})^2 \end{aligned}$$

2 $$\begin{aligned} IAA_{img,c}= & {} \sqrt{(1-ssd_{c})} * \dfrac{\max (\hat{n}, n-\hat{n})}{n} \end{aligned}$$

where $p_{ic}$, $p_{jc}$ are the serving size annotations of annotators *i*, *j*, respectively for food category *c*. The number of annotators that annotated the specific category is $\hat{n}$ and the total number of annotators is *n* (where $\hat{n}\le n$). $ssd_{c}$ plays the role of disagreement between the annotators for food category *c* and the term $[\dfrac{n(n-1)}{2}]^{-1}$ normalizes its value between 0 and 1. The operator $\max (\cdot )$ results in the number of annotators that annotated *c*, if they are more than the annotators that did not annotate it and vice versa. $IAA_{img,c}$ is the *IAA* for the specific image for category *c* and $\overrightarrow{IAA_{c}}$ is a vector that contains the $IAA_{img,c}$ for all images that include category *c*. The total *IAA* for all the images and all categories *C* is then defined as the weighted average for all categories, based on the times the category appears in the dataset $(N_{i})$:

3 $$\begin{aligned} Total_{IAA} = \dfrac{\sum _{i=1}^{C} N_{i} * mean(\overrightarrow{IAA_{c}})}{\sum _{i=1}^{C}N_{i}} \end{aligned}$$

**Figure 2:**
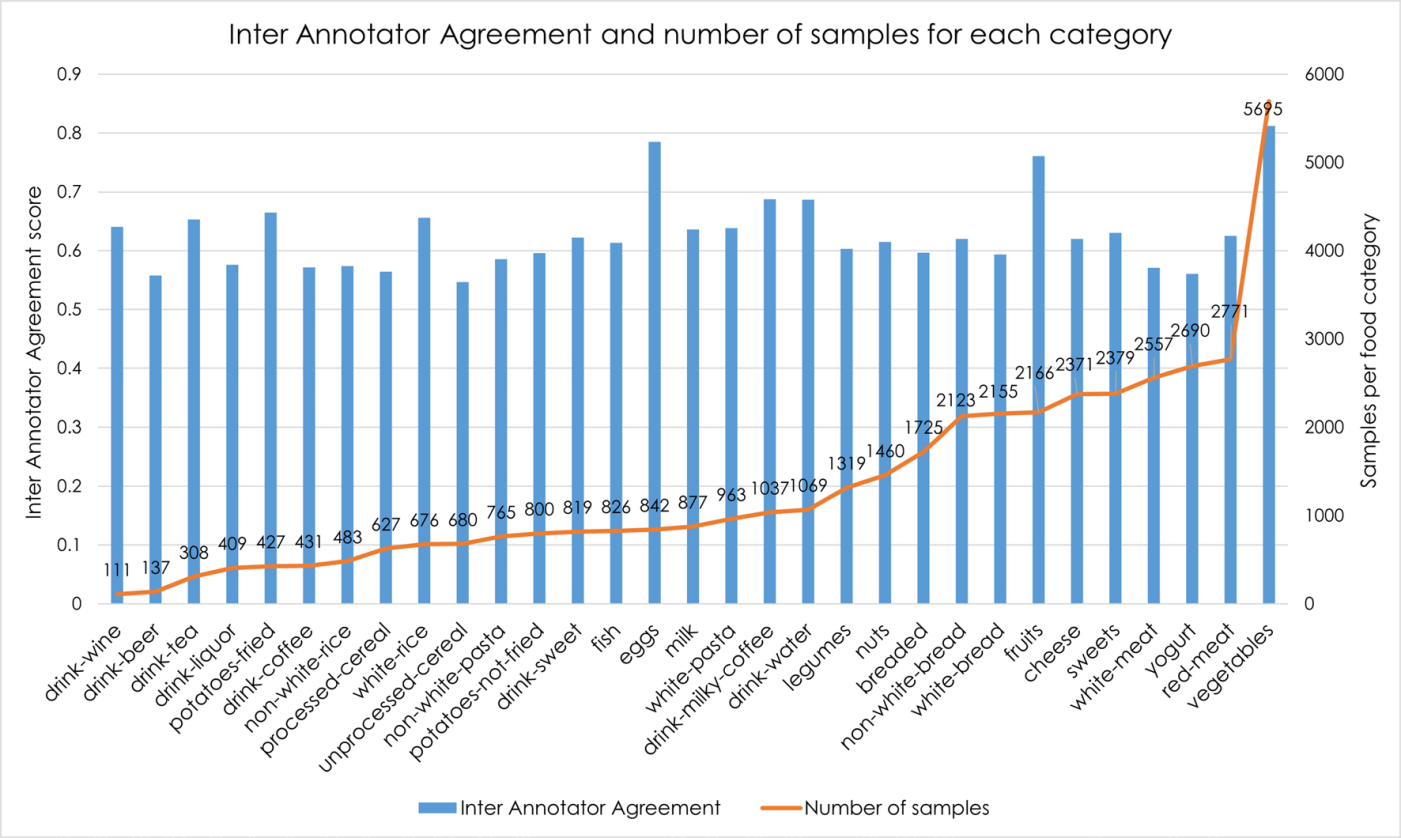
Inter annotator agreement $(IAA_{c})$ for each of the 31 food categories, ranked from the category that has the fewest samples in the training set (wine) to the one that has the most (vegetables).

We calculated the IAA for each category, for the 9104 images annotated by 5 annotators (Fig. 2). In the figure, the categories are ranked in ascending order of the frequency of their samples in the training set. The $Total_{IAA}$ for the entire annotated dataset is equal to 64.7%, demonstrating that the training dataset contains label noise.

### Food recognition and serving size estimation

In this section, we initially present the network architecture that is used to perform food item recognition and serving size estimation. However, the presence of label noise in the training dataset can heavily deteriorate the results of the food recognition and serving size estimation tasks, since CNNs tend to “learn” from noisy labels and generalise poorly on a clean testing set^22^. Therefore, a proper method that can consider the label noise of the dataset is required. In^23^ and^24^ a noise-adaptation layer is appended to a neural network (NN) to learn the distribution between the noisy labels and the true, hidden ones. Other methods^25,26^ rely on a small, free-of-noise subset that can help with learning from noisy data. Moreover, there are methods that require the training of two NNs and each one separates the clean from the noisy samples that are going to be used from the other NN^27,28^. In this section, we also explain the noise-robust training procedure that is going to be used.

For the network architecture, we use the same architecture as described in our previous work^4^. We use (a) a CNN to extract features from images and (b) the pre-trained GloVe^29^ to extract word semantic features. A Graph CNN (GCNN)^4,30^ that uses these features and the correlation between the different food items is trained to recognize the food categories and their serving sizes.

For the training procedure, we adapt the methodology of^27,31^ for the multi-label problem. Specifically, two networks are trained simultaneously, and each model divides the dataset into a clean and a noisy subset to be used by the other model. Then, in order to counter the label noise, the samples are interpolated with each other based on^32^ so that the models learn to behave linearly between training samples.

Based on the work of DivideMix^27^, two NNs are initially trained for a few epochs (“warmup”). This way, two individual NNs can make predictions without overfitting to the label noise.

A Gaussian Mixture Model (GMM) is then fit on the per-sample loss of each network to divide the dataset into a clean set and a noisy-unlabeled set based on a fixed threshold. The two subsets will be used from the other network, to avoid error accumulation. At each epoch, there are two iterations where one model is being trained, while the other is being fixed. Initially, both the clean and the noisy sets are augmented by using random crops and horizontal flips on the images. For the noisy set, the labels are being replaced by the average of predictions from both networks on the augmentations as in (4), while for the clean set, the labels are refined based on their probability of being clean (5):

4 $$\begin{aligned} Y_{noisy}= & {} \dfrac{1}{2M}\sum _{m}(p_{1}(U_{m}) + p_{2}(U_{m})) \end{aligned}$$

5 $$\begin{aligned} Y_{clean}= & {} w_{clean}y_{clean} + (1-w_{clean})\dfrac{1}{M}\sum _{m}p_{1}(L_{m}) \end{aligned}$$

where *M* is the number of augmentations, $p_{1}$ is the model to be trained, $p_{2}$ is the fixed model, $U_{m}$ and $L_{m}$ are the noisy and the clean subsets respectively, $w_{clean}$ are the probabilities of the labeled samples being clean, and $y_{clean}$ are the original labels of the clean subset. $Y_{noisy}$ and $Y_{clean}$ refer to the final labels of the noisy ($X_{noisy}$) and the clean ($X_{clean}$) subset, for both the food category and the serving size estimation.

In the end, the data are further augmented^27,31,32^. Specifically, for each sample *i* from a batch *b*, the augmented image, the corresponding labels, and the serving sizes are mixed as follows:

6 $$\begin{aligned} z'_{i,b}=\lambda z_{i,b} + (1-\lambda )z_{j,b} \end{aligned}$$

where *z* is either the augmented image, the label, or the serving size, *j* is a random sample from the batch, and $\lambda$ is a random sample from the beta distribution ($\lambda >0.5)$. This way, the networks are trained to give linear predictions between samples, even if the labels are noisy.

The augmented input data are then fed into the network to be trained. Since there are two targets we are trying to optimize, we use a) the binary cross entropy loss for the mixed clean labels, b) the mean squared error loss for the mixed noisy labels and the serving sizes. In the beginning, the loss from the noisy set is discarded, as the models are not ready to predict the noisy labels, but gradually its weight is increased as the training procedure progresses.

The model with the best performance was integrated into the end-to-end automated MDA adherence system which automatically performs food recognition and serving estimation and outputs the MDA score on a weekly basis, along with suggestions for a healthier diet, closer to the MD.

We used the ResNet-101 model^33^, pre-trained on ImageNet^34^, as the feature extractor. We used the Adam optimizer with a learning rate of $10^{-4}$ and a batch size of 32 for the ”warmup” stage for 5 epochs and a learning rate of $10^{-5}$ and batch size of 12 for the remaining 10 epochs. We used a threshold of 0.5 for the output of the GMM to distinguish the clean from the noisy subset and $M = 2$ augmentations for each input image. We also used a loss weight of 1 for the labels and 0.1 for the serving sizes throughout the procedure since the prediction of the food categories is more important.

### Mediterranean diet adherence score

The weekly MDA score can be calculated based on food items that are consumed on an (a) meal, (b) daily, and (c) weekly basis^3^. A set of rules has been defined by expert dietitians^4^ and are being further refined here. Firstly, the 31 food categories that the network predicts must be clustered into 13 coarser categories, which share similar nutritional values, namely: vegetables, fruits, cereal, nuts, dairy products, alcoholic beverages, legumes, fish, white meat, red meat, eggs, sweets, and potatoes. Apart from these categories, we also consider olive oil, which plays a major role in the MD. While we use our automated food recognition system for the recognition of most of the food types, identifying the use of oil used in the preparation of a food is extremely challenging. Hence, we provide the option to the user to manually enter this category and use this for the weekly MDA scoring.

(a) Meal-based Score: Fruits, vegetables, and olive oil add plus points when they are consumed within any meal (breakfast, lunch, dinner, or snack), while cereal adds to the score only when consumed as a part of the main meals (breakfast, lunch, dinner). For each of these food categories, the scoring is summed for the whole day with a maximum scoring of 3/7 points per day (Supplementary Table 1).

(b) Daily-based Score: Nuts, dairy products, and alcoholic beverages are not related to meals, but give points if they are consumed throughout the day (Supplementary Table 2).

(c) Weekly-based Score: For the food categories that are counted on a weekly basis (legumes, fish, eggs, white meat, red meat, sweets, potatoes), the servings are summed up for the whole week to give the respective points (Supplementary Table 3).

The food categories that are scored on an (a) meal and (b) daily basis are summed for the whole week and added to the (c) weekly-based scoring to give the final weekly MDA score. The score lies from 0 (no adherence to MD) to 24 (highest adherence to MD).

We then adapt the score using (7). The $MDA_{0-100}$ score is then normalized between 0% and 100% so it can be interpreted easier (Supplementary Fig. 1) and a small increase in the original score would be mapped to a higher increase in the $MDA_{0-100}$ score, encouraging participants to follow a healthier diet.

7 $$\begin{aligned} MDA_{0-100} = (\ln (MDA_{0-24} +1))^2 \end{aligned}$$

### Smartphone application

The smartphone application consists of an interface which allows end-users to collect images of their daily meals and annotate them. Using the smartphone application, a user can capture a photo of a meal/food item. The user can also select the meal type and, optionally, choose the food categories that appear in the image, to be used for validation purposes (Supplementary Fig. 2). However, annotating olive oil in the image is crucial for the MDA scoring since it is not automatically recognized. While we highly encouraged the users participating in the study to take photos of their meals, they were provided with the option to also log only a textual description of their meals and annotate the MDA categories present in the meal. Once the users’ images are uploaded to the Oviva AG^35^ platform, an end-to-end system runs the food recognition and serving size estimation algorithms and applies the MDA rules to calculate the weekly MDA score for the patient. At the end of each week, the system sends out a detailed report to each user regarding their weekly MDA score. The report consists of four parts:

1. An MD Explainer which reminds the user the key points of the MD.
2. A colored percentage weekly score of their MDA (Supplementary Fig. 3).
3. A traffic light system regarding certain food categories important to the MD. If they were on track with a category, it was marked as Green, while categories, which needed further improvement were marked either in Yellow or Red (Supplementary Fig. 3).
4. Detailed recommendations on how to improve the MDA score for each food category. These recommendations are provided only for the categories, which had traffic lights displayed as Red or Yellow.

### Feasibility study

**Figure 3:**
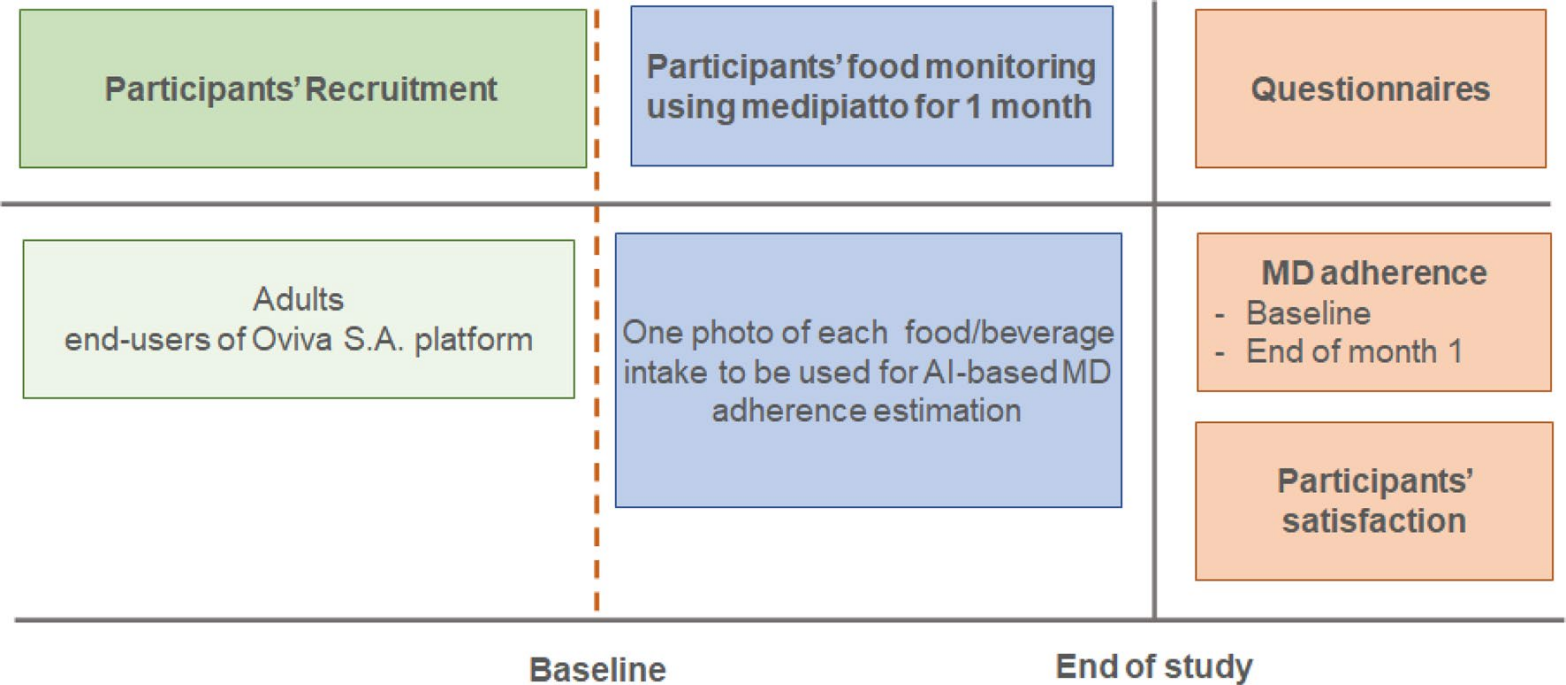
The layout of the feasibility study.

The outline of the feasibility study is shown in Fig. 3. The goal was to recruit at least 20 end-users of the Oviva AG platform (Body Mass Index $>27\hbox { kg/m}^{2}$). The study consisted of three stages: (i) the baseline, or the trial preparation stage, (ii) the duration of the trial, which involved the participants’ food monitoring using the medipiatto system for 1 month, and (iii) the end of the study, which involved the calculation of the self-reported MDA of the participants and the obtaining of answers to trial evaluation questionnaires that were handed out to both participants and the dietitians who recruited them.

During the baseline stage, the users were asked to fill out a 15-item validated food frequency questionnaire (FFQ) to assess their self-reported MDA score and collect information about their current food intake and dietary habits, based on a previous study^9^. The self-assessment questionnaire is a multiple-choice questionnaire with each question contributing points, to a total score of 30 (Supplementary Table 4). The participants also reported their sex, age, height, weight, highest level of educational attainment, current employment status, and nationality (demographics).

During the trial stage, the participants had to use the newly introduced system for a period of one month. They were asked to take photos of their food/beverage intakes using the mobile app and optionally, annotate the food categories. At the end of each week, the participants received their percentage MDA score, a traffic light color system that demonstrates their scores on 8 important to MD food categories, an explanatory sheet regarding the MD, and suggestions to improve their MDA score. The 8 food categories that we chose to present are fruits, vegetables, cereals, nuts, legumes, fish, red meat, and sweets, since a slight change in their consumption can be easily observed in the weekly MDA score.

Finally, at the end of the study, after a period of one month from the start, the participants were asked to fill out the same 15-item questionnaire to assess their self-reported MDA score, as well as a qualitative feedback questionnaire regarding their satisfaction with using the system. A qualitative feedback questionnaire was also administered to the dietitians treating the participants.

### Statistical analysis

All analyses were performed using the SciPy library of Python. To measure statistical significance, we performed the paired t-test. Statistical significance was considered at the value of P<0.05.

### Ethics approval and consent to participate

The study was reviewed and declared exempt from ethics review by the Cantonal Ethics Committee, Bern, Switzerland (KEK, Req-2021- 00225). All the participants were informed about the project and signed an informed-consent form. They had the option to drop out of the study at any time and have their data removed if they wanted to. All research was performed in accordance with relevant guidelines/regulations and the principles of the Helsinki Declaration.

## Results

### Dietary assessment

To evaluate our system for the food recognition task, we adopt the mean Average Precision (mAP) metric, that is widely used for multi-label classification problems. For the evaluation of the system’s ability to estimate the serving size of the food items in a meal, we used the mean Absolute Percentage Error (mAPE).

**Figure 4:**
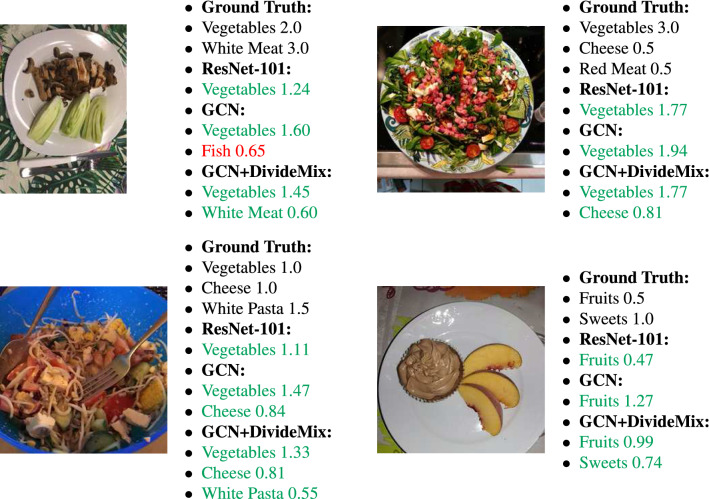
Results from the three methods for the food recognition and the serving size estimation. Categories appear in green and red for correct and wrong predictions respectively.

Table 2 shows a comparison of the mAP and the mAPE for the simple ResNet-101 model, the GCN architecture from our previous work^4^, and the GCN architecture following the DivideMix training procedure described in this paper. As we can see, even though the GCN model provides worse results than the baseline ResNet-101 model for the mAPE, the noise-robust GCN outperforms both. It achieves an mAP of 61.8% and an mAPE of 54.5%. It is worth mentioning, that the GCN model following the DivideMix training procedure, has better AP for the 7 out of the 10 most common food categories, while for the other 3, the difference is less than 3%. The classes with the worst AP score are breaded food, unprocessed cereal, non-white rice, and legumes which all have less than 1000 samples in the training set and/or share visual and nutritional similarities with other categories (e.g., white rice, processed cereal). Figure 4 shows some experimental results from the different methods.

**Table 2:** Comparison of mean average precision (mAP—higher is better) and mean absolute percentage error (mAPE—lower is better) between the ResNet-101, the GCN, and the GCN architecture with noise-robust training procedure.

	mAP	mAPE	Time (ms)
ResNet-101	0.565	0.584	17
GCN	0.603	0.605	20
GCN + DivideMix	**0.618**	**0.545**	20

We also compared the results for the MDA score of the system versus a nutrition expert performing visual estimations for 4 users. The expert’s estimations for the MDA score for the four users are 63%, 42%, 55%, and 48% while the system’s estimations are 57%, 37%, 57%, and 49% respectively, which corresponds to a mean difference of only 3.5% between their estimations.

### Feasibility study

Thirty-five users were approached by the dietitians to be a part of the study. Among them, thirty-two users expressed interest and agreed to participate in the study. Four participants decided to drop out of the program before the completion of 4 weeks. Two participants logged, on average, less frequently than 3 times per week and cited personal reasons for this and, therefore, were not effectively considered in the study. Further two participants did not respond to the final questionnaire. We ended up with 24 participants (21 female, 3 male) who participated in the study, regularly logged meals, and provided a response to the final questionnaire. Additional information regarding their demographics can be seen in Table 3.

**Table 3:** Participants’ demographics (n = 24).

Characteristic	Value
Sex (n, %)	Female (n = 21, 87.5%)
	Male (n = 3, 12.5%)
Mean age in years (SD)	46.9 (13.1)
Mean starting BMI in kg/m^2^ (SD)	31.8 (4.4)
Nationality (n, %)	Swiss (n = 22, 91.7%)
	Italian (n = 1, 4.15%)
	German (n = 1, 4.15%)
Highest level of educational attainment (n, %)	Technical high school (n = 3, 12.5%)
	High school (n = 14, 58.4%)
	Higher technical college (n = 2, 8.3%)
	Bachelor (n = 1, 4.15%)
	Master’s degree (n = 3, 12.5%)
	PhD (n = 1, 4.15%)
Current employment status (n, %)	Employed (n = 16, 66.7%)
	In further training (n = 1, 4.15%)
	Retired (n = 3, 12.5%)
	Full-time mothers (n = 3, 12.5%)
	Not specified (n = 1, 4.15%)

At the end of each week, the system performed image analysis on the meal images captured by every participant in order to recognize the food items that appear in them, as well as their serving sizes. A total of 2072 food images were acquired, which corresponds to an average of  3.1 images per day, per participant. The system predicted that an average of 2.25 food items appear in each image. Vegetables was the most common food category logged, appearing 850 times in the resultant image dataset, followed by fruits and yoghurt.

**Figure 5:**
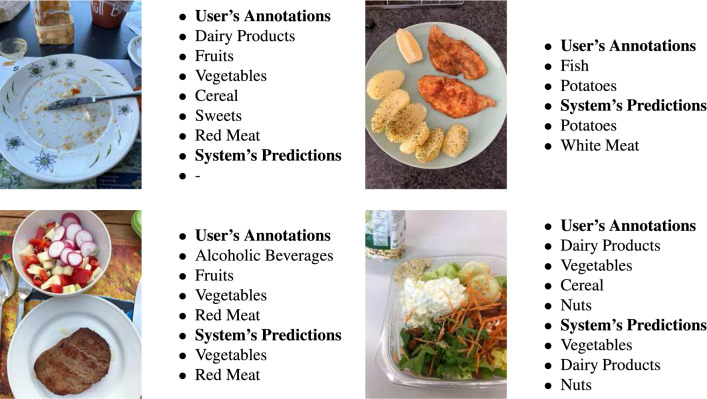
Images acquired from the feasibility study, along with the users’ annotations.

While capturing a photo of their meal, the users also had the option to annotate the images with the food categories they consumed. The users annotated approximately 50% of their images. Comparing the system’s predictions against the user-provided annotations, the mAP was 57.3%. The fact that the mAP was lower than that of the original testing dataset is sensible, since a lot of the images were not annotated properly. A common scenario was that the users annotated all the food items they consumed in the meal, even though only a few of those were visible in the image. Example images from the feasibility study, as well as the users’ manual annotations are shown in Fig. 5. In the testing set of the initial database that was used to evaluate the system’s performance, an expert dietitian cleaned out its labels, while the images acquired from the feasibility study contained a lot of label noise, and were evaluated without cleaning.

Table 4 shows the weekly system-estimated MDA scores for each participant. The estimations are based on the logged images of the participants or on the annotated labels in case the user did not post a photo. In the case where there are only annotations but no image, the serving size is estimated as the average of the serving sizes for the specific food category. There are nine cases where the MDA score increased, two cases where the MDA score remained the same, and 13 cases where the MDA score decreased. This could be due to the fact that participants were more motivated in the beginning of the study and logged more frequently compared to the $4^{\mathrm{th}}$ week. We can calculate the per meal average MDA score by dividing the final weekly MDA score by seven times the average number of logs per day. In this case, out of the 24 participants, 18 had an increase in their average meal score between the first and the fourth week (column Average Meal Score of Table 4).

**Table 4:** % MDA scores per participant for each week (Wi, i = 1,. . ., 4), as calculated by the system and the FFQ.

Participant number	Weekly % MDA score (average logs/day)					Average meal score	FFQ score
	W1	W2	W3	W4	W4–W1	W4–W1	W4–W1
1	33 (2.3)	36 (2.4)	34 (2.3)	30 (1.3)	$-$ 3	8.7	$-$ 2
2	45 (6.1)	28 (2.9)	37 (2.7)	37 (3.0)	$-$ 8	5.0	$-$ 3
3	39 (2.9)	45 (2.6)	45 (3.0)	54 (3.3)	+ 15	2.9	1
4	45 (4.9)	24 (0.3)	28 (0.9)	31 (0.7)	$-$ 14	33.8	2
5	47 (2.6)	34 (2.3)	33 (1.3)	22 (0.4)	$-$ 25	36.9	1
6	48 (2.0)	37 (2.1)	41 (2.1)	40 (1.9)	$-$ 8	$-$ 2.9	5
7	42 (3.0)	37 (2.9)	48.0 (3.3)	43 (2.4)	+ 1	3.9	$-$ 3
8	61 (5.6)	56 (5.4)	50.0 (4.6)	60 (4.1)	$-$ 1	3.7	5
9	42 (2.1)	60 (3.0)	45.0 (2.4)	40 (2.3)	$-$ 2	$-$ 2.6	2
10	50 (3.9)	49 (3.1)	51.0 (3.4)	54 (2.3)	+ 4	10.7	1
11	42 (1.3)	40 (2.4)	37.0 (1.6)	39 (1.9)	$-$ 3	$-$ 11.8	8
12	47 (3.1)	41 (3.4)	46.0 (3.1	54 (2.6)	+ 7	5.6	$-$ 1
13	51 (3.4)	49 (3.3)	43.0 (3.3)	45 (2.7)	$-$ 6	1.7	5
14	51 (4.6)	52 (5.6)	55.0 (4.7)	54 (4.4)	+ 3	0.4	5
15	29 (3.7)	50 (3.6)	39.0 (3.7)	40 (3.0)	+ 11	5.5	4
16	55 (3.0)	51 (3.1)	51.0 (3.0)	48 (2.7)	$-$ 7	$-$ 0.6	2
17	41 (7.1)	47 (6.6)	42.0 (6.6)	41 (5.6)	0	1.5	3
18	50 (4.0)	53 (2.4)	43.0 (3.1)	56 (3.9)	+ 6	2.2	5
19	49 (2.9)	48 (3.0)	51.0 (3.3)	43 (2.6)	$-$ 6	$-$ 0.4	1
20	60 (3.0)	62 (3.0)	55.0 (2.9)	57 (2.7)	$-$ 3	1.1	3
21	37 (2.0)	28 (1.6)	28.0 (1.1)	37 (1.1)	0	15.1	$-$ 2
22	47 (3.0)	54 (3.7)	35.0 (1.7)	37 (1.7)	$-$ 10	6.1	5
23	46 (2.1)	49 (2.9)	49.0 (2.9)	49 (2.6)	+ 3	$-$ 3.1	1
24	43 (2.9)	48 (2.7)	57.0 (3.0)	59 (2.6)	+ 16	7.9	6
Average	45.8 (3.4)	44.9 (3.1)	43.5 (2.9)	44.6 (2.6)	$-$ 1.25	5.5	2.25

The average number of images logged per day, for every week is shown in parentheses for W1–W4. The weekly % MDA score (W4–W1) and the average meal score (W4–W1) columns show the difference between the last and the first week for the MDA score and the average meal score respectively. The final column shows the difference between the final and initial FFQ scores.

#### Self-reported food frequency questionnaire

The self-reported FFQ (Supplementary Table 4) is used here as a self-measurement of a person’s compliance with the MD. The mean and standard deviation of the questionnaire response scores at the beginning of the study were $17.3\pm 3.4$. The mean and standard deviation of the self-reported response scores at the end of the study were $19.5 \pm 3.3$ (column FFQ Score of Table 4). Out of the 24 participants, 19 participants had an overall increase in the score calculated from the self-reported FFQ, while five participants showed a decrease in the score. There was a significant difference in the scores at the beginning and at the end of the study ($t = -3.68, p=0.001$). In general, retired participants and full-time mothers tend to log more frequently than employees (3.6 logs/day compared to 2.9 logs/day). In addition, retired participants had the highest average weekly MDA scores as estimated by both the system and the self-reported questionnaire (54.5% MDA score, 19.8/30 self-reported score), followed by full-time mothers (49.0 % MDA score, 18.5 self-reported score), and employees (43.5 % MDA score, 18.1 self-reported score).

#### Qualitative feedback questionnaire on the app usage: participants

Out of the 26 participants that successfully completed the study, 24 participants responded to the questionnaire. 20 participants (83%) responded positively that they would be willing to continue to integrate the recording and weekly evaluation of the MD as a part of their everyday life. The participants who responded negatively pointed out that in their opinion the score was not accurate, and the suggestions were not personalized and actionable. The majority of participants (23/24) reported that the system was easy to use on a daily basis. Three participants suggested that the recording system could be improved by adding the possibility of logging their meals using an audio recording (number of participants, n = 2) or by making a video (n = 1). Some participants reported that the following food groups were misinterpreted by the used system in the traffic light report: cereal (n = 4), fruits (n = 4), vegetables (n = 4), nuts (n = 2), pulses (n = 1), sweets (n = 1), and red meat (n = 1). The traffic light system was negatively pointed out as not being helpful by eight participants, while five participants did not have an opinion about it. The time frame of the weekly report was qualified as positive by 23 participants, while one participant opted for a longer period of two weeks.

#### Qualitative feedback questionnaire on the app usage: dietitians

All five dietitians reported that the use of the MD in the study could be advantageous to their weight loss consultation. 4 out of 5 dietitians agreed that providing participants with improvement tips in the weekly report can be motivating for them to change their dietary habits and that food logging is supporting the positive outcomes of their participants. All five dietitians agreed that the MD is suitable for the treatment of weight-loss participants. However, the dietitians reported that often the calculated scores were difficult to interpret by the participants and that the participants felt that the food images were sometimes misidentified by the food recognition algorithm. Regarding a feasible duration for the participants to log their meals daily, three dietitians opted for a time period of up to one month; the remaining two reported that more than one month can be done in practice as well.

## Discussion

In this study, we validated the performance of a fully automatic end-to-end system that recognizes the food/drink items that appear in a single meal image, estimates their serving sizes, and calculates the user’s weekly MDA score. The database that was used for the training of the system contained natural label noise, since it was annotated by inexperienced annotators. We quantified the total inter-annotator agreement, which was equal to 64.7%. For this reason, we used the GCN network of our previous work^4^ and adapted the noise-robust training procedure of^27^ for a multi-label problem. Compared to a baseline model (ResNet-101) and the GCN, the GCN following the noise-robust training procedure achieves better results in both food item recognition and serving size estimation, with an mAP and mAPE of 61.8% and 54.5% respectively. As for the computation time for a single input, the difference between the ResNet-101 model and the GCN models is negligible, while for the 2 GCN models, the computational time is the same since they share the same network architecture.

As an evaluation of our system, an expert in health and nutrition manually annotated the weekly meal logs of 4 subjects regarding the food/drink items that appear in the photos, as well as their serving sizes. We then used the MDA formula to calculate the % MDA score for the 4 participants. The results of the system and the expert were very close to each other; a mean absolute error of 3.5% was achieved.

For the feasibility study, we recruited 24 participants that followed all the necessary steps. The participants had to fill out a validated self-reported FFQ in the beginning and in the end of the study, as well as a qualitative feedback questionnaire after a period of one month. We observed that for the self-reported FFQ most of the participants (19/24) had an increase in their MDA score, while for the system’s estimations only 9/24 participants had their MDA score increased. This can be mainly attributed to the fact that the self-reported questionnaire is vague and that the participants tend to log fewer meals as the study goes on. 20/24 participants reported that they would be interested in using the application for the MDA and 21/24 reported that the daily recording was straightforward and self-explanatory. The five dietitians responsible for the participants had to also fill out a qualitative feedback questionnaire. The feedback from the dietitians was positive, and 4/5 dietitians reported that the weekly MDA score could motivate the users towards a healthier lifestyle. In another pilot study^36^ that took place in Spain, participants were recruited to use a nutritional education app that offers MD-based dietary counseling. It was found that the participants had a significant increase in carbohydrate intake and a significant decrease in fat intake in a period of one month and improved their dietary habits significantly in three months. However, the application uses only manual input from the user and does not include any AI-based functionalities for dietary assessment.

However, there are still a few limitations to our study. The sample size is too small to draw significant conclusions regarding the effect of MD and any health implications that this could have for a user. The study period was also rather short to observe a significant change in a participant’s dietary habits. Moreover, even though the participants found the app user-friendly and easy-to-use, they tend to be more motivated and log more frequently in the beginning rather than at the end of the study. Therefore, additional ways of increasing the engagement with the user should be considered. Finally, the majority of the participants were female and, thus, a more equal representation needs to be taken into account in future studies so as to observe the impact of app usage according to sex.

Regarding our future steps, we firstly intend to conduct a clinical trial with a larger number of participants coming from different countries and for a longer study period, in order to investigate the effects of the system on the MDA and the BMI of the participants. For this reason, additional data to train the food recognition and serving size estimation network will be acquired, that cover a wider range of cuisines, while the network will also be able to learn based on the users’ new data. The period of usage of such apps needs to be investigated so as to optimally design studies that depict user needs. HCPs’ and users’ perspectives on the usability and acceptability of an optimized version of our system need to be considered. In addition to the MDA score, which will be further enhanced so that it is easier to achieve higher scores, even in non-Mediterranean countries, we will also consider adapting the score to cover other healthy diets (e.g., rich in protein, low carbohydrate), so that the users will be able to select the diet that best fits their needs and preferences. Finally, a comparison of our innovative system with some conventional dietary assessment methods has to be conducted to test its effectiveness as a stand-alone solution.

## Conclusion

In this manuscript, we present an AI-based, end-to-end system that automatically performs food recognition, serving size estimation, and MDA score calculation and accordingly provides tailored feedback to the user. The food image analysis is done by a GCN that is trained using a noise-robust training procedure, which outperformed the baseline method in both food recognition and serving size estimation tasks. We have conducted a feasibility study to evaluate the performance of our AI-powered system in quantifying the adherence of a person with the MD and assess its usability, functionality, and the effect of such a system on the participants’ dietary habits. 24 participants took part in the study, where they had to capture photos of their meals for 4 weeks. The results from the qualitative feedback questionnaire from the participants and the dietitians showed that they were satisfied with the app usage.

## Supplementary Information

Supplementary Information.

## Data Availability

The datasets generated and/or analysed during the current study are not publicly available due to privacy constraints but are available from the corresponding author on reasonable request and with the permission of Oviva AG.
