# Supplementary material for: A feasibility study to assess Mediterranean Diet adherence using an AI-powered system
Source: Sci Rep. 2022 Oct 11;12:17008. doi: 10.1038/s41598-022-21421-y (PMC9554192; doi:10.1038/s41598-022-21421-y)
Supplement: Supplementary file 1 — Supplementary Information. [file 41598_2022_21421_MOESM1_ESM.pdf]

A feasibility study to assess Mediterranean Diet Adherence using an AI-powered system

Ioannis Papathanail[1], Maria F. Vasiloglou[1], Thomai Stathopoulou[1], Arindam Ghosh[2], Manuel Baumann[2], David Faeh[3], and Stavroula Mougiakakou[1,*]

[1]ARTORG Center for Biomedical Engineering Research, University of Bern, Murtenstrasse 50, Bern, 3008, Switzerland
[2]Oviva AG, Zürcherstrasse 64, Altendorf, 8852, Switzerland
[3]Epidemiology, Biostatistics and Prevention Institute (EBPI), University of Zurich, Zurich, 8001, Switzerland
[*]stavroula.mougiakakou@unibe.ch

Supplementary Information

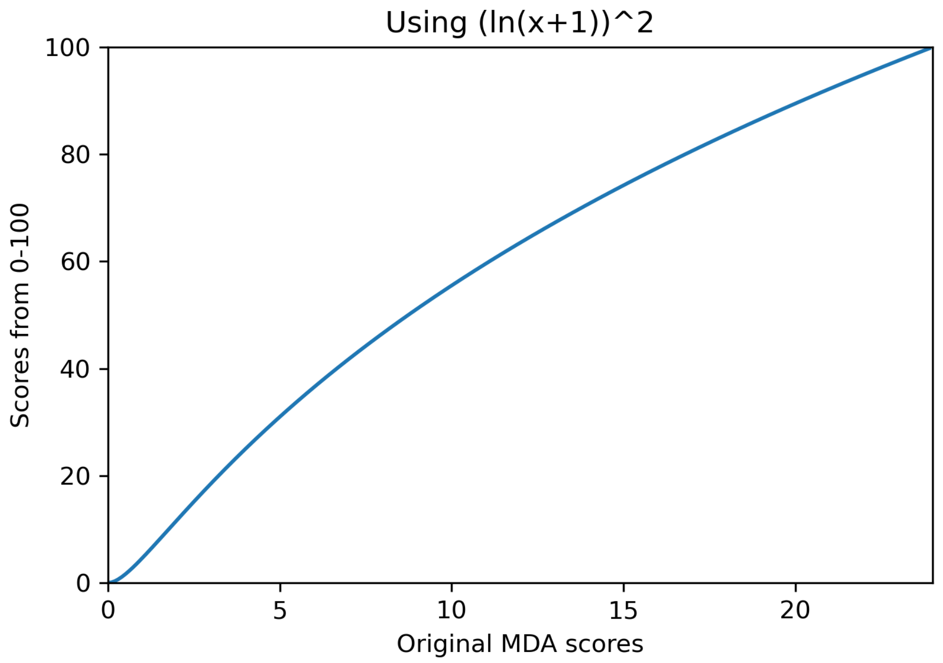

Figure 1. Function of transforming the old into the new MDA score that ranges from 0% to 100%

Table 1. The MDA scoring for food groups counted on a meal basis (Max 3/7 per day for each category)

Category	Meal	Servings per WEEK	Point
Fruits	Any	0	0
		≥ 1	1/7
Vegetables	Any	0	0
		1	1/7
		≥ 2	2/7
Cereal	Main meals	0	0
		≥ 1	1/7
Olive Oil	Any	0	0
		≥ 1	1/7

Table 2. The MDA scoring for food groups counted on a daily basis

Category	Meal	Servings per WEEK	Point
Nuts	Any	0	0
		≥ 1	2/7
Dairy Products	Any	0	0
		1	1/7
		2-4	2/7
		≥ 5	1/7
Alcoholic Beverages	Any	≤ 2	1/7
		> 2	0

Table 3. The MDA scoring for food groups counted on a weekly basis

Category	Meal	Servings per WEEK	Point
Legumes	Any	< 2	0
		≥ 2	1
Eggs	Any	< 2	0
		2-7	1
		≥ 8	0
Fish	Any	< 2	0
		≥ 2	1
White Meat	Any	< 2	0
		≥ 2	1
Red Meat	Any	≥ 2	0
		< 2	1
Sweets	Any	> 2	0
		≤ 2	1
Potatoes	Any	> 3	0
		≤ 3	1

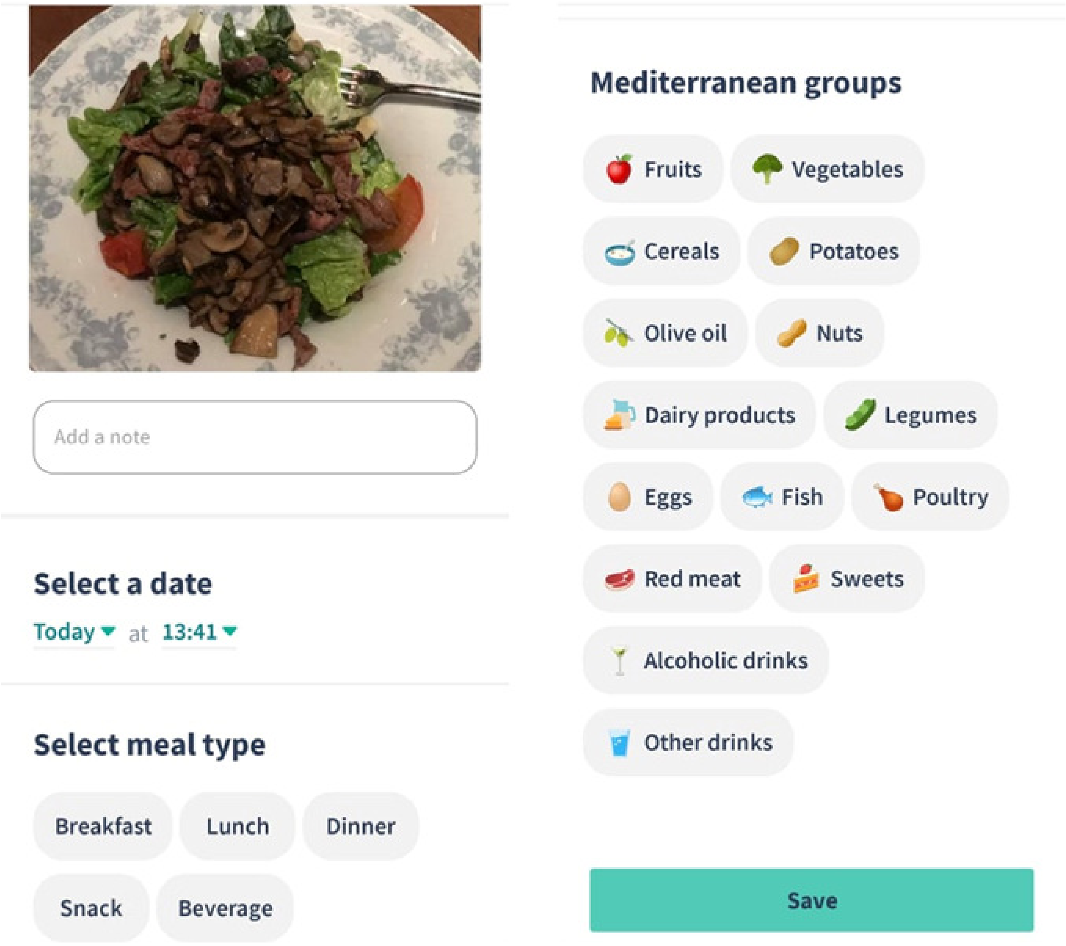

Figure 2. Screenshots of the Oviva app

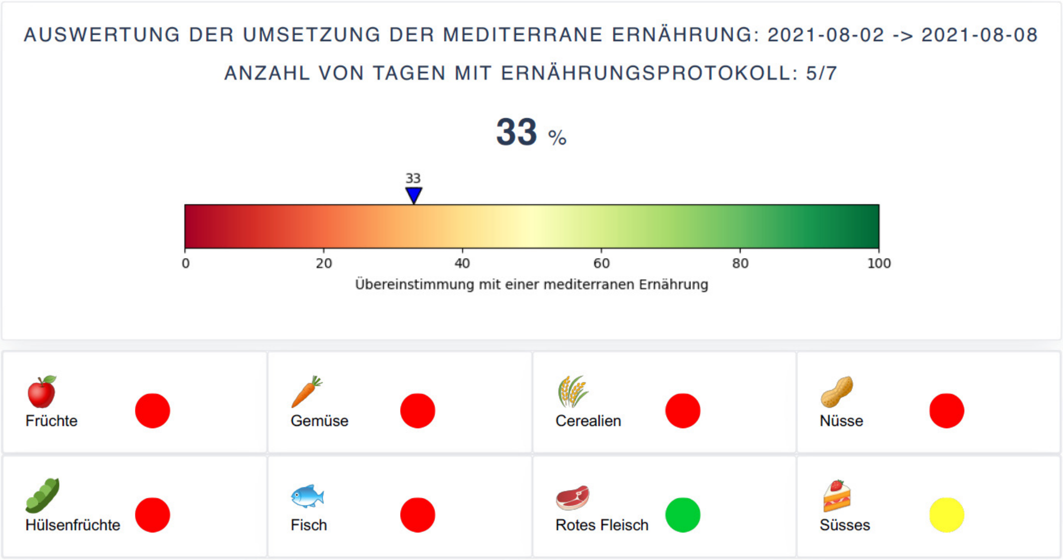

Figure 3. Percentage weekly MDA score and traffic lights for important MD-related food categories

Table 4. The 15-question MDA food frequency questionnaire

Nr.	Question	Answer					
			Pt		Pt		Pt
1	Do you mainly use rapeseed or olive oil for cooking?	Yes, (almost) every day	2	Yes, 2-4 days a week	1	Less than 2 days per week	0
2	Do you mainly use rapeseed or olive oil for salads?	Yes, (almost) every day	2	Yes, 2-4 days a week	1	Less than 2 days per week	0
3	Do you eat cooked vegetables as a side dish?	Yes, (almost) every day	2	Yes, 2-4 days a week	1	Less than 2 days per week	0
4	Do you eat raw vegetables as a snack or salad?	Yes, (almost) every day	2	Yes, 2-4 days a week	1	Less than 2 days per week	0
5	Do you eat fresh fruit?	Yes, (almost) every day	2	Yes, 2-4 days a week	1	Less than 2 days per week	0
6	Are you eating a red meat or sausage dish?	Yes, (almost) every day	0	Yes, 2-4 days a week	1	Less than 2 days per week	2
7	Do you include bread with butter for breakfast and/or dinner?	Yes, (almost) every day	0	Yes, 2-4 days a week	1	Less than 2 days per week	2
8	On how many of the past 7 days did you drink a cola and/or other beverage sweetened with sugar?	6 to 7 days	0	3 to 5 days	1	2 days or less	2
9	Do you pay attention to the dietary fiber content in your diet (whole grain instead of normal variant)?	Yes, (fairly) consistently	2	Yes, occasionally	1	No, practically not	0
10	How many times a week do you eat legumes such as lentils, chickpeas or beans?	3 or more days per week	2	1 to 2 days a week	1	Practically never	0
11	How many days a week do you serve fish or seafood?	2 or more days per week	2	1 day per week	1	Less than 1 day per week	0
12	How often per week do you eat at least a handful of raw, unsalted nuts?	3 or more days per week	2	1 to 2 days a week	1	Practically never	0
13	Would you choose chicken, turkey, or rabbit rather than beef, pork, hamburgers, or sausage?	Yes, (almost) always	2	Rather yes	1	No, probably or definitely not	0
14	Does your menu include steamed vegetables, pasta, rice or other dishes served with a tomato, garlic, onion or leek sauce sautéed with canola or olive oil?	Yes, several times a week	2	Yes, about 1 time per week	1	No, practically never	0

15	Have you eaten anything breaded or fried in the past 7 days?	Yes, in 3 or more days	0	Yes, in 1 to 2 days	1	No	2
